# Molybdenum Deficiency Produces Motor Nervous Effects That Are Consistent with Amyotrophic Lateral Sclerosis

**DOI:** 10.3389/fneur.2016.00028

**Published:** 2016-03-08

**Authors:** Christopher A. Bourke

**Affiliations:** ^1^Veterinary Diagnostic Laboratory, Orange Agricultural Institute, Orange, NSW, Australia

**Keywords:** amyotrophic lateral sclerosis, motor neuron disease, molybdenum deficiency, endogenous purines, sulfur amino acids

Amyotrophic lateral sclerosis (ALS)–motor neuron disease (MND) is divisible into 6 familial and 10 sporadic syndromes (1). Familial syndromes are also recognized in animals, namely, dogs, cattle, rats, and mice. This article is concerned with the most common form of ALS, sporadic Charcot ALS (SC-ALS). This form is of particular interest because it does not occur in any animal as a complete disease; instead, each of its three parts can occur as separate syndromes in sheep, horses, and cats. It is possible that physiological differences between these animals and humans could explain this separation. In addition, an understanding of the etiology of each animal syndrome could reveal a common predisposing factor that is responsible for generating the complete disease. Molybdenum (Mo) deficiency has recently been demonstrated as the predisposing factor for two of the animal syndromes and the third has a pathogenesis that is consistent with Mo deficiency. This raises the possibility that Mo deficiency is the predisposing factor for SC-ALS.

Two of the three animal syndromes referred to occur in sheep and are associated with Mo deficiency. Both produce specific motor nervous effects not known to occur in any other sporadic, adult onset, animal disorder. The first is xanthosine motor neuron syndrome (XMNS) (2). In this chronically progressive and irreversible syndrome, an asymmetric muscle weakness develops in one pelvic limb and sometime later, in the corresponding thoracic limb. The muscle weakness is accompanied by atrophy, and dysfunction is restricted to particular extensor muscles. The commonest presentation of SC-ALS is similar to this and involves asymmetric weakness and atrophy of some muscle groups in either upper or lower limb. Asymmetric muscle weakness in mammals is produced by a very precise upper motor dysfunction that can only emanate from the basal ganglia in animals, such as sheep, and from the basal ganglia and motor cortex in humans (3–5). Xanthosine ingestion during Mo deficiency is the only etiology so far demonstrated in a mammal, which can produce this type of progressive and irreversible asymmetric muscle weakness.

The second Mo deficiency-associated sheep disorder is inosine motor neuron syndrome (IMNS) (6). In this chronically progressive and irreversible syndrome, a brain stem-generated bulbar muscle weakness develops together with a brain stem-generated respiratory muscle weakness. These two dysfunctions lead to difficulty in vocalizing, swallowing, and breathing, including a reduced forced vital capacity.

In SC-ALS, 30% of cases present with bulbar signs and in the remaining 70% bulbar signs usually develop during the course of the disease. Bulbar involvement in SC-ALS is often closely correlated with respiratory muscle weakness, including reduced forced vital capacity (1).

Inosine ingestion during Mo deficiency is the only etiology so far demonstrated in a mammal, which can produce this type of progressive and irreversible muscle weakness. The lingual, laryngeal, and pharyngeal muscle dysfunctions that cause the bulbar effects can be generated by either abnormal upper motor control over the hypoglossal nuclei in the medulla or by abnormal functioning of the hypoglossal nuclei themselves. The diaphragm and intercostal muscle dysfunction that causes the respiratory effect can be generated in two different ways: first, *via* abnormal upper motor control over the pontomedullary respiratory center, which then affects the phrenic nerve neurons in the cervical spine; or second, *via* abnormal activity of the cervical phrenic nerve neurons themselves. In IMNS, the former is the case, whereas, in SC-ALS, it is more likely that that the former occurs first but the latter develops later on.

The third part of SC-ALS that occurs in animals has only been observed in horses (7) and cats (8). It is referred to as equine and feline MND, respectively. Both appear to be clinically and pathologically similar but much more is known about the former (EMND) than the latter. EMND is a sporadic, idiopathic, lower MND. It occurs in adult horses that have been eating an unbalanced diet for over 12 months or more. Similar to SC-ALS, this disease is characterized by the development of profound, bilaterally symmetrical muscle weakness, atrophy, and fasciculations, which affect the head, neck, axial skeleton, and limbs.

In EMND, these motor nervous effects are caused by the degeneration of the lower motor neuron affiliated neurons in the brain stem and the spinal motor neurons in the ventral (anterior) horns. In SC-ALS similar effects occur and are caused by the same neuronal degeneration. It is noteworthy that in EMND there is no report of asymmetric limb muscle weakness, bulbar muscle weakness, or respiratory muscle weakness. The cause of EMND is thought to be dietary but no specific imbalance has been demonstrated, so far. Low levels of the antioxidant, vitamin E, are frequently identified in affected horses (9, 10). Based on the reasoning that follows, Mo deficiency could be the predisposing factor for this disorder and presumably for that part of SC-ALS that equates with it.

Molybdenum deficiency predisposes mammals to CNS disease because it inactivates both xanthine oxidase-dehydrogenase (the enzyme barrier that protects the CNS from dietary purine loading) and sulfite oxidase (the enzyme barrier that protects the CNS from dietary sulfite loading). Mo is an essential cofactor for these two enzymes. XMNS and IMNS are caused by purine loading, and EMND could potentially be caused by sulfite loading. Dietary sources of the purine nucleosides xanthosine, inosine, adenosine, and guanosine, and their bases xanthine, hypoxanthine, adenine, and guanine, respectively, can cause purine loading, and dietary sources of sulfur amino acids can cause sulfite loading. Purine loading leads to astrocyte degeneration (2, 6), and this adversely affects normal astrocytic modulation of neurotransmission. Sulfite loading blocks the normal conversion of methionine to cysteine, and this causes homocysteine values to rise and cysteine values to fall (11). Elevated homocysteine favors motor neuron degeneration (12), and depressed cysteine favors glutathione deficiency, hence greater susceptibility to motor neuron degeneration (13). When the antioxidant glutathione becomes less available, reserves of the antioxidant, vitamin E, will be drawn upon. Physiological differences among sheep, horses, and humans in the way they process purines and sulfur amino acids, render sheep and humans susceptible to purine loading, but not horses, and horses and humans susceptible to sulfite loading, but not sheep. Purine loading occurs in species that both salvage and excrete purines, namely, sheep and humans. It does not occur in species that only excrete purines, namely, dogs, cattle, rats, and mice. Hence, it is necessary to use a sheep model rather than a mouse model for these studies. Likewise, sulfite loading occurs in mongastric species, such as humans and horses, but not in ruminant species, such as sheep and cattle; hence, sheep are an unsuitable model for studies of this kind.

The author proposes that Mo deficiency in humans makes them susceptible to the potential neurotoxic effects of dietary xanthosine, inosine, and sulfur amino acids, and it is the combined effect of these three substances that produces SC-ALS. The following circumstantial evidence supports the notion that a large number of humans are Mo deficient, hence, at risk of this disease. The daily Mo intake per person in the US is 0.18 mg (14) and in the UK is 0.11 mg (15). If daily food intake is 3% of body weight and the average body weight is 83 kg, then the Mo content of the average US diet would be 0.07 ppm, and the average UK diet 0.04 ppm. By comparison, Mo deficiency is known to produce motor nervous disease in sheep when the Mo content of the diet is <0.04 ppm (range <0.01–0.20) (2). On this basis, a large number of US and UK citizens are Mo deficient. In the US, the incidence of ALS is 1.87 per 100,000 (16) and in the UK it is 3.25 per 100,000 (17). The prevalence figures for each country also reflect this difference, approaching 4 per 100,000 in the US and 7 per 100,000 in the UK. The 61% lower Mo intake in the UK is therefore associated with a 74% higher incidence of ALS, and this is consistent with Mo deficiency predisposing to ALS. The low blood urate levels commonly recorded for patients with ALS offers further support for this opinion (18). Low blood urate levels are indicative of low xanthine oxidase activity, and low xanthine oxidase activity is caused by Mo deficiency.

One final aspect of Mo deficiency requires some comment. Mo deficiency can potentially produce both astrocyte degeneration and neuron degeneration. The contribution that neuron degeneration makes to the pathogenesis of SC-ALS is universally agreed upon; however, there is also an emerging awareness of the potential contribution of astrocyte degeneration to this disease (19). Both XMNS and IMNS have demonstrated that astrocyte degeneration can generate progressive and irreversible motor nervous effects and that this process involves a very long preclinical latent period. It is possible that SC-ALS also involves a very long preclinical latent period, which would be consistent with its normal age of onset of >55 years, and that astrocyte degeneration plays a part in this. In the subset of ALS that occurs on the Pacific island of Guam, it has been possible to follow individuals who have left Guam without clinical disease, not returned, and then gone on to develop ALS. From two such studies, the period between leaving Guam and developing ALS has been determined to be 1–34 years with a mean of 14 years (20, 21). Sheep studies have demonstrated that Mo deficiency precipitated motor nervous effects develop after a mean preclinical latent period of 2 years. Assuming a life expectancy of 91 years for humans and 13 years for sheep, the 2-year mean latent period in sheep would equate to 14 human years. Considering that the causal agent is already loaded by the time the preclinical latent period commences, it is difficult to accept that neuronal degeneration starts on day 1 of the latent period and yet clinical effects do not appear for another 14 years. It would make more sense if a more subtle process such as astrocyte degeneration was initiated on day 1 of the latent period and neuronal degeneration not until many years after that day.

There is an accumulation of evidence suggesting that some sporadic cases of ALS are at least partly due to genetic mutations (22). In the light of this evidence, it is possible that rather than Mo deficiency in isolation being the cause of SC-ALS it could be that Mo deficiency is the environmental factor that leads to the complete disease in persons of a particular genotype.

## Author Contributions

The author confirms being the sole contributor of this work and approved it for publication.

## Conflict of Interest Statement

The author declares that the research was conducted in the absence of any commercial or financial relationships that could be construed as a potential conflict of interest.
